# Usability and Acceptability of a Text Message-Based Developmental Screening Tool for Young Children: Pilot Study

**DOI:** 10.2196/10814

**Published:** 2019-01-30

**Authors:** Pamela Ryden Johnson, Jessica Bushar, Margaret Dunkle, Sharon Leyden, Elizabeth T Jordan

**Affiliations:** 1 Voxiva, Inc Arlington, VA United States; 2 Child Health Foundation Timonium, MD United States; 3 ZERO TO THREE Washington, DC United States; 4 Department of Health Policy School of Public Health George Washington University Washington, DC United States; 5 Prince George's County Health Department Clinton, MD United States; 6 College of Nursing University of South Florida Tampa, FL United States

**Keywords:** developmental screening, EPSDT, ITP, low income, Medicaid, mHealth, mobile health, Text4baby, text messaging, PEDS:DM, WIC

## Abstract

**Background:**

Only 30% of parents of children aged 9-35 months report that their child received a developmental screening in the previous year. Screening rates are even lower in low-income households, where the rates of developmental delays are typically higher than those in high-income households. Seeking to evaluate ways to increase developmental screening, *Text4baby*, a national perinatal texting program, created an interactive text message-based version of a validated developmental screening tool for parents.

**Objective:**

This study aimed to assess whether a text message-based developmental screening tool is usable and acceptable by low-income mothers.

**Methods:**

Low-income mothers of infants aged 8-10 months were recruited from the Women, Infants and Children Program clinics in Prince George’s County, MD. Once enrolled, participants used text messages to receive and respond to six developmental screening questions from the Parents’ Evaluation of Developmental Status: Developmental Milestones. After confirming their responses, participants received the results and feedback. Project staff conducted a follow-up phone survey and invited a subset of survey respondents to attend focus groups. A representative of the County’s Infants and Toddlers Program met with or called participants whose results indicated that their infants “may be behind.”

**Results:**

Eighty-one low-income mothers enrolled in the study, 93% of whom reported that their infants received Medicaid (75/81). In addition, 49% of the mothers were Hispanic/Latina (40/81) and 42% were African American (34/81). A total of 80% participated in follow-up surveys (65/81), and 14 mothers attended focus groups. All participants initiated the screening and responded to all six screening questions. Of the total, 79% immediately confirmed their responses (64/81), and 21% made one or more changes (17/81). Based on the final responses, 63% of participants received a text that the baby was “doing well” in all six developmental domains (51/81); furthermore, 37% received texts listing domains where their baby was “doing well” and one or more domains where their baby “may be behind” (30/81). All participants received a text with resources for follow-up. In a follow-up survey reaching 65 participants, all respondents said that they would like to answer screening questions again when their baby was older. All but one participant would recommend the tool to a friend and rated the experience of answering questions and receiving feedback by text as “very good” or “good.”

**Conclusions:**

A mobile text version of a validated developmental screening tool was both usable and acceptable by low-income mothers, including those whose infants “may be behind.” Our results may inform further research on the use of the tool at older ages and options for a scalable, text-based developmental screening tool such as that in *Text4baby*.

## Introduction

Less than one-third (30.4%) of the parents of children aged 9-35 months in the United States surveyed report that their child received a developmental screening in the previous year [1]. Screening rates are even lower among young children in low-income families and Medicaid, where the prevalence of developmental delays is higher than among children in higher income and privately insured families [2].

Low developmental screening rates among young children persist despite well-established benefits of early identification of developmental delays (when intervention may be most effective) [3,4]; the American Academy of Pediatrics (AAP) recommendations for periodic screening of young children (coinciding with the 9-, 18-, and 24- or 30-month well-baby visits) [4,5]; the fact that timely developmental screening is a core quality measure for children’s health and a required benefit for children under 3 years of age in Medicaid as part of Early and Periodic Screening, Diagnostic and Treatment, which provides comprehensive and preventive health care services for children and adolescents [6,7]; ready availability of validated paper-based and online developmental screening tools [5,6,8]; and the fact that early development lays the very foundation for a child’s growth and development and “lifelong trajectory” [7,9].

This pilot study explored an innovative use of mobile phones and text messaging to expand the availability of validated, parent-reported developmental screening, especially among low-income populations covered by Medicaid. The use of mobile phones and text messages may be a particularly effective way to reach young families at risk, since 100% of adults between the ages of 18 and 29 years and 92% of all adults with incomes below $30,000 surveyed by the Pew Research Center in January 2018 reported that they owned a cell phone of some kind [10]. Further, in as early as 2011, a Pew survey found that “95% of 18-29 year olds use the text messaging feature on their phones” [11].

This pilot study addresses several gaps in the current literature. First, although paper-based and online developmental screening tools are well documented and evaluated [6,8,12], to our knowledge, there are no published assessments of the use of validated text messages by parents to report on the developmental milestones. In one study, as part of the Baby Steps program, researchers conducted a user study of 14 Hispanic families who received shortened versions of the Ages & Stages screening questions by text message [13]. The researchers found that parents liked the text-based tool and based on the user responses, concluded that “text messaging is a feasible tool for supporting parents in tracking and monitoring their child’s development” [13]. A recent systematic review of multiple systematic reviews of mobile health (mHealth) interventions (including text messaging) included no references to studies of developmental screening but concluded that text message reminders have a consistent impact on public health outcomes such as appointment attendance and improved treatment adherence for some chronic conditions [14].

Second, although the need for developmental screening is most acute among underserved, low-income populations, according to a recent review article, overall “research regarding the use of mHealth interventions for the populations that need it most remains sparse” [15]. This review [15] also cites the study partner *Text4baby* [16] as an exception that specifically targets low-income pregnant women and mothers of infants receiving Medicaid [15]. Since its launch in 2010, the free national text messaging service *Text4baby* has delivered more than 430 million messages with health and parenting tips (G Perez-Bonany, personal communication, Dec 2018), appointment reminders, and health surveys corresponding with the due date of the mother or the age of the infant [17,18].

Third, although there are many examples of assessments of the usability and acceptability of web-based health interventions, there are very few rigorous assessments of the usability of mHealth technologies [19]. This pilot study adapted measures from the Health IT Usability Evaluation Model, which is based on experience with the evaluation of Web-based programs and tested with several mHealth programs [19].

The aims of this pilot study were to build and assess the usability and acceptability of a text-based validated developmental screening tool for infants of low-income mothers as part of a plan to implement the lessons learned in a large-scale deployment and trial, potentially via an existing mHealth service such as *Text4baby*. Three hypotheses guided the study: (1) It is technically feasible to create a text-based version of an existing validated developmental screening tool; (2) low-income mothers will find text-based developmental screening usable, regardless of their infants’ developmental status; and (3) low-income mothers whose infants “may be behind” may find using the tool and receiving immediate feedback about their infants’ developmental status less acceptable than mothers who receive feedback that their infants are “doing well.”

The pilot study also aimed to determine whether infants who “may be behind” in one or more developmental domain could be followed up after the mother received the results of the screening. The initial findings are presented here; additional findings and lessons concerning the follow-up and maternal actions following the screening will be presented in a subsequent paper.

## Methods

### Screening Tool

The study team built a text-based developmental screening tool on the *Text4baby* technology platform based on Parents’ Evaluation of Developmental Status: Developmental Milestones (PEDS:DM), a validated paper- and Web-based screen [12]. The team then worked with the Maryland Department of Health and Mental Hygiene (MDHMH); Prince George’s County’s Women, Infants and Children (WIC) Program; and Infant and Toddlers Program (ITP) to pilot the tool with low-income mothers of infants aged 8-10 months. The MDHMH Institutional Review Board approved the study.

### Technical Feasibility of a Text-Based Developmental Screening Tool

A number of validated tools are used to conduct developmental screening; most are paper-based tools and generally administered in a provider’s office, daycare center, or other site. The most current list of accepted tools is presented in the 2018 Technical Specifications for the Child Core Set of health quality measures for Medicaid and the Children’s Health Insurance Program [6]. AAP also provides links to validated screening tools and supporting documentation [5]. Although some of these tools have been adapted for online administration, to our knowledge, none are currently available in a text format.

Our study team selected its screening tool following a review of three validated tools [8] (Multimedia Appendix 1) that are also cited by AAP and Medicaid [5,6]. All three tools are characterized by the coverage of multiple mental, behavioral, and developmental domains; appropriateness for infants below the age of 12 months and through early childhood; consistency with AAP recommendations; use of parental responses based on their own observations; and accuracy and strong validation studies referenced in the review (>70% specificity and sensitivity) [8].

Of these three validated tools, the PEDS:DM is the most amenable to adaptation to an interactive text format. Each of the PEDS:DM questions for the target age is less than 160 characters long, so each question could be sent in a single text message (Table 1). Further, the PEDS:DM covers the six developmental domains (fine motor, expressive language, receptive language, gross motor, self-help, and social-emotional) with only six questions, each written at an elementary grade reading level [8,20]. In contrast, Ages & Stages [8] uses 30 questions written at the 3rd-12th grade level, a number of which exceed the 160-character limit of a text message and would require editing for length as well as reading level to be suitable for use in a text-based tool appropriate for the target audience.

The study team worked with PEDS:DM to incorporate the questions and responses (Table 1) and feedback (Table 2) in a text format, build the tool on the *Text4baby* technology platform, and check the text version for quality. Neither the wording of the PEDS:DM questions nor the scoring criteria were altered.

PEDS:DM is generally administered in a provider’s office by staff or self-administered on paper or online. The study team developed a plan with the ITP of Prince George’s County to ensure that follow-up was offered in person or by phone to mothers whose infants’ results indicated that they “may be behind.”

### Recruitment and Enrollment of Low-Income Mothers

The study team was able to reach and recruit low-income mothers by partnering with the Maryland WIC program, an income-qualified program “designed to help low-income pregnant, postpartum, and breastfeeding women, infants, and children 5 years old or younger who are at nutritional risk” [21]. The Maryland WIC program defines low income as US $37,296 annually for a family of three people [21].

Staff in two WIC clinics in Prince George’s County offered a flyer about the study to mothers of infants aged 8-10 months who were visiting one of the clinics for services. Interested mothers were invited to a private area of the clinic where members of the study team described the study, answered questions, and advised the mothers that after completing the screening, they would receive a US $20 gift card and a gift bag with information about their baby’s development. The study team members then confirmed the eligibility of interested mothers to participate in the study. To be eligible, a mother needed to be the primary caretaker of an infant aged 8, 9, or 10 months; above the age of 18 years; able to speak and read English; and in possession of a mobile phone that was regularly used, including for texting. Eligible mothers filled out a form providing informed consent and were enrolled as participants in the study. After enrollment, the study team members collected information on participant and infant characteristics including age, gender, insurance status, and ethnicity/race (Table 3).

### Usability

To measure usability, the study used objective data generated by the technology platform that reflected efficiency and effectiveness (Table 4). Study participants were asked to initiate the interactive screening tool by texting “DM” to the *Text4baby* short code 511411; this generated an automatic reply with the first of the PEDS:DM screening questions, asking the participants to respond by text message. Each time a participant texted a response to a question, the platform automatically sent a text message with the next question. After participants answered all six screening questions, a study team member asked them to review the questions and their responses. If they wished to make changes, study staff asked participants to text “DM” again to retake the screening questions.

Following any changes made, participants confirmed their answers by texting “RESULTS.” In response, they received a message listing areas where their infant appeared to be “doing well” and, if indicated, a second message with any areas where he/she “may be behind” (Table 2). The mothers then received a final text message with recommended actions. Following the screening, study team members also provided participants with a letter containing the results intended for the infants’ primary pediatrician from the Infants and Toddlers Program (under Part C of the Individuals with Disabilities Education Act) or a qualified child care provider, a US $20 gift card, and a gift bag that included a guide for parents on child development [22].

The *Text4baby* technology platform captured system data on measures of participant interaction. These data included a record of all questions and responses and date and time stamps for all outgoing and incoming texts.

### Acceptability

To measure acceptability, beginning two weeks after the screening, study staff called all mothers and administered a phone survey (Table 5) to those who replied. This phone survey included questions related to participants’ experience using the text-based PEDS:DM and receiving feedback about their baby’s development via text.

Two subsets of mothers who participated in the phone survey were invited to attend one of two focus groups—one included seven mothers whose infants were “doing well” in all domains and the other included seven mothers whose infants “may be behind” in one or more domain. An independent evaluator facilitated the focus groups. Participants received a gift card to cover transportation and other costs. The aim of the focus groups was to provide context and insight into the usability and acceptability of the tool and learn about maternal actions following the screening (to be presented in a subsequent paper).

### Analysis

Usability and acceptability were examined among participants overall and by the developmental screening status of their infants. Applicable systems and phone survey data were used to assess each measure. In addition, observations made by mothers in the focus groups that related to usability and acceptability were included. A coding scheme was applied to qualitative data from interviews and focus groups to identify key themes.

Descriptive analyses were used to analyze quantitative systems and phone survey data. Where appropriate, quantitative analysis was performed using the Chi-square test. In many of the cases, there were too many zero cells, and therefore, statistical analysis could not be performed.

## Results

### Technical Feasibility

It was possible to take the existing validated PEDS:DM questions and program them on the *Text4baby* technology platform (Tables 1 and 2). Because the screening questions were already at a low reading level (second grade) and short (under 160 characters), it was not necessary to make any changes to their wording. Furthermore, because the response options were closed, simple, and clear (ie, “no,” “a little,” and “yes”), it was only necessary to add instructions (eg, “Reply 1 for no”). There were no changes in the thresholds for determining risk. The study team devised additional text messages to facilitate administration, deliver results, and provide appropriate feedback for the participants.

**Table 1:** Developmental screening tool: questions and response options. Responses in italics indicate that the baby is meeting the milestone.

Topic of message	PEDS:DM^a^ questions for children aged 8-10 months	Response options	Text version	Characters (including spaces), n
Fine motor	Can your baby poke at things with just his/her first finger?	No, A little, *Yes*	Can your baby poke at things with just his/her first finger? Reply 1 for No, Reply 2 for A little, Reply 3 for Yes.	115
Expressive language	When you say your baby’s name, does he or she stop and look at you?	No, Sometimes, *Most of the time*	When you say your baby’s name, does he or she stop and look at you? Reply 1 for No, Reply 2 for Sometimes; Reply 3 for Most of the time.	136
Receptive language	How many different sounds, such as “muh,” “bah,” “duh,” or “guh” does your baby say?	None, One, *Two or more*	How many different sounds, such as “muh,” “bah,” “duh,” or “guh” does your baby say? Reply 1 for None, Reply 2 for One, Reply 3 for 2 or more.	142
Gross motor	Can your baby get around on hands and knees or by scooting on his or her bottom?	No, *Sometimes*, *Yes*	Can your baby get around on hands and knees or by scooting on his or her bottom? Reply 1 for No, Reply 2 for Sometimes, Reply 3 for Yes.	136
Self-help	Does your baby try to get to toys that are out of reach?	No, A little, *Yes*	Does your baby try to get to toys that are out of reach? Reply 1 for No, Reply 2 for A little, Reply 3 for Yes	111
Social emotional	Does your baby like to play peek-a-boo?	No/Never tried, *A little/Yes*	Does your baby like to play peek-a-boo? Reply 1 for No/Never tried, Reply 2 for A little/Yes.	93

^a^PEDS:DM: Parents’ Evaluation of Developmental Status: Developmental Milestones

**Table 2:** Developmental screening tool: feedback

Feedback	Results in text	Characters (including spaces), n
For mothers reporting 1/more met milestones	Based on your answers, your baby does well [using hands and fingers, listening, talking, using arms and legs, learning to take care of self, getting along with others]	Varied
For mothers reporting 1/more unmet milestones	Based on your answers, your baby may be behind [using hands and fingers, listening, talking, using arms and legs, learning to take care of self, getting along with others]	Varied
For mothers reporting all milestones met	Thanks, Mom! Keep up with how your baby is learning and growing. Go to www.pgcitp.net^a^ or call 301 265-8415 if you have questions.	128
For mothers reporting 1/more unmet milestones	Please call Prince George’s Infants & Toddlers right away at 301 265-8415 for FREE help for your baby. Talk with baby’s Dr. & childcare provider too.	148

^a^Website currently unavailable.

### Participants’ Characteristics

A total of 81 low-income mothers of infants aged 8-10 months were recruited and enrolled in the study: 93% (75/81) of the mothers reported that their infants were on Medicaid, 2% (2/81) had no insurance, 4% (3/81) had private insurance, and 1% (1/81) did not know her child’s insurance status (Table 3). Seventeen percent (14/81) of the mothers said that they had less education than a high school degree, 37% (30/81) had a high school degree, and 46% (37/81) had some college or higher education.

Nearly half of the mothers (49%, 40/81) indicated that they were Hispanic/Latina, 42% (34/81) identified as African American, and 5% (4/81) identified as white (non-Hispanic). In addition, 57% of the participants (46/81) listed English, 37% (30/81) listed Spanish, and 6% (5/81) listed another language as the first language spoken at home.

Of note, 15 mothers (19%) reported that their infant had been born prematurely compared to a statewide prematurity rate of 10.5% among all women and 12.5% among African American women in Maryland [23].

Most participants (91%, 74/81) reported using a mobile phone with internet access, and 9% (7/81) reported using a basic phone without internet access. Ninety percent of the participants (73/81) reported that they had a monthly texting plan that provided unlimited text messaging, 5% reported a monthly plan with limited texting (4/81), 1% (1/81) paid on a per-message basis, and 4% (3/81) did not know what their texting plan provided.

There were no statistically significant differences between subsets of these low-income mothers for any of the demographic and other characteristics except previous screening. Mothers who reported that their infant had been previously screened were significantly more likely to have infants who were “doing well” in comparison with those who did not report a previous screening (*P*<.02). Overall, 47% of the mothers reported that their infant previously received a similar screening (38/81); this proportion is higher than the national average and the Maryland statewide rate of 43% [1].

### Usability

The assessment of usability of the screening tool focused on five objective measures of efficiency and effectiveness: initiation of the screening tool, completeness of responses to screening questions, time required, ability to receive results, and follow-up of infants who “may be behind” (Table 4).

Several mothers had connectivity issues with their phones inside one of the clinics but were able to connect by moving closer to a window. One of the mothers received a message from her carrier that the carrier would charge her for text messages. Since the study protocol specified that messages would be free, the study team lent her an alternative phone to use for the study.

All participants—mothers whose infants “may be behind” or were “doing well”—were able to text “DM” to the short code 511411 on their mobile phones (100%, 81/81). All were able to initiate the screening and successfully trigger the first question. In addition, all were able to trigger and enter one of the indicated responses to each of the six questions (100%, 81/81). A total of 79% (64/81) successfully submitted responses to all six questions on their first attempt. The remaining 21% (17/81) submitted final responses on the second or, in the case of one mother, third time. Three of these participants required additional time because of technology/connectivity issues. Four restarted the tool during submission of the six responses because they made an error in one or more responses.

After entering all responses to the last question, a study team member asked all participants to review their responses. Among the fourteen participants who changed one or more of their responses when they answered the questions for a second time, 86% (12/14) changed their response to Question 1 (“Poke”). Response to Question 2 (“Baby’s Name”) was the second most frequently changed: Half of those who changed their responses (7/14) changed their response to this question.

One focus group participant described her confusion about the meaning of “poke” as follows:

I got kind of confused when they said poking. Poking, how? What does that mean? I had to re-read it again. I was thinking—Poke what?

Additionally, although only three participants changed their answers to Question 6 (“Peek-a-boo”), some expressed concern about this question in the focus groups:

Peek-a-boo? I did not understand. I had a hard time understanding what it was all about. Actually, that my baby was doing that.

**Table 3:** Demographic and other characteristics of participants and their infants.

Characteristics		All participants (N=81)	“Doing well” (N=51)	“May be behind” (N=30)	*P* value for “Doing well” vs “May be behind”
Mother’s age (years), mean (SD)^a^		27.6 (5.7)	27.9 (5.5)	27.1 (6.1)	.53
**Mother’s education, n (%)**					
	Less than high school	14 (17)	10 (20)	4 (13)	.18
	High school graduate	30 (37)	15 (29)	15 (50)	—^b^
	Some college or higher	37 (46)	26 (51)	11 (37)	—
**Mother’s race/ethnicity, n (%)**					
	Non-Hispanic/African American	34 (42)	23 (45)	11 (37)	.86
	Hispanic/Latina^c^	40 (49)	24 (47)	16 (53)	—
	Non-Hispanic, white	4 (5)	2 (4)	2 (7)	—
	Other	3 (4)	2 (4)	1 (3)	—
**Infant’s gender, n (%)**					
	Female	41 (49)	29 (57)	12 (40)	.14
	Male	40 (51)	22 (41)	18 (60)	—
**Language spoken at home, n (%)**					
	English	46 (57)	28 (55)	18 (60)	.70
	Spanish	30 (37)	19 (37)	11 (37)	—
	Other (eg, Amharic or French)	5 (6)	4 (8)	1 (3)	—
**Infant born prematurely, n (%)**					
	Yes	15 (19)	9 (18)	6 (20)	.79
	No	66 (81)	42 (82)	24 (80)	—
**Infant’s source of health insurance, n (%)**					
	Medicaid	75 (93)	49 (98)	26 (87)	.30
	No health insurance	2 (2)	1 (2)	1 (3)	—
	Private insurance	3 (4)	1 (2)	2 (7)	—
	Don’t know	1 (1)	0 (0)	1 (3)	—
**Type of text messaage plan, n (%)**					
	For each text message	1 (1)	0 (0)	0 (0)	.12
	Monthly for unlimited texts	73 (90)	47 (92)	27 (90)	—
	Monthly for a limited number of texts	4 (5)	1 (2)	3 (10)	—
	Don’t know	3 (4)	3 (6)	0 (0)	—
**Screening status^d^, n (%)**					
	All 6 milestones met	48 (59)	—	—	—
	5 or fewer milestones met	33 (41)	—	—	—
**Final screening status^e^, n (%)**					
	All 6 milestones met	51 (63)	—	—	—
	5 or fewer milestones met	30 (37)	—	—	—
**Previous screening of infant, n (%)**					
	Yes	38 (47)	30 (59)	8 (27)	<.02
	No	40 (49)	20 (39)	20 (67)	—
	Missing	3 (4)	1 (2)	2 (7)	—

^a^One participant did not provide her age.

^b^Not applicable.

^c^Mothers who provided another related term for race (eg “Spanish” or “Guatemala”) or who did not respond to the question were identified as Hispanic/Latina if they also indicated that they spoke Spanish at home.

^d^As submitted by mothers via the screening tool.

^e^In 3 cases, the mother indicated that she had made a mistake on a single question after she had received the results, which changed the infant’s screening status. The final screening status reflects the amended responses in these cases. This status is reflected in the letter prepared for the baby’s provider, a copy of which was also given to the mother.

**Table 4:** Usability of a text-based developmental screening tool.

Measures of efficiency and effectiveness				Overall (N=81)	“Doing well” (N=51)		“May be behind” (N=30)
**Efficiency, n (%)**							
	Initiation of tool (participants able to text “DM” to 511411 and trigger developmental screening questions)			81 (100)	51 (100)		30 (100)
	**Responding to screening questions**						
		Participants able to trigger and respond to all 6 developmental screening questions		81 (100)	51 (100)	30 (100)	
		**Attempts required to complete all 6 developmental screening questions**					
			Completed on first attempt	64 (79)	—^a^		—
			Completed on second attempt	16 (20)	—		—
			Completed on third attempt	1 (1)	—		—
	Time required for completion (average amount of time to complete all 6 screening questions), min			4.39	4.38		4.4
**Effectiveness, n (%)**							
	Receiving results (participants receiving results by text following completion of screening questions),			81 (100)	51 (100)		30 (100)
	**Follow-up of infants with one/more missed milestones: Did the mother of infant with one/more missed milestone meet/talk with ITP^b^ program?**						
		ITP representative met with mother (only)		—	—	3 (10)	
		ITP representative met with and reached the mother by phone		—	—	9 (30)	
		ITP representative reached the mother by phone (only)		—	—	16 (53)	
		ITP representative called but was not able to reach mother (did not meet with)		—	—	2 (7)	

^a^Not applicable.

^b^ITP: Infants and Toddlers Program.

To follow-up with the 30 mothers whose responses indicated that their infants “may be behind,” the study team took a number of steps that will be described in detail in a forthcoming paper: All 30 mothers received a text message encouraging them to call the ITP. On the days when an ITP representative was present in the WIC clinic during the study, she met with any mother whose infant had one or more unmet milestones. She also attempted to call all these mothers to administer the follow-up survey. By the end of the study, the ITP representative had met or talked with all but two of the mothers (93%, 28/30).

### Acceptability

Acceptability of the screening tool was assessed by a phone survey by using subjective measures of ease of use, usefulness, and satisfaction (Table 5). It was possible to reach and interview 80% of study participants (65/81). Almost all interview participants said that it was “Easy” (40%, 26/65) or “Very Easy” (58%, 38/65) to answer the six text message developmental screening questions; none said it was “Hard” or “Very Hard” (Table 5). As described by one focus group participant, “I have two kids and I’m on the phone or busy with kids. It’s easier to answer the questions on a phone than to have to talk on the phone.” A higher percentage of the participants whose infants were “doing well” said the text messages were “Very Easy” (70%, 28/40), compared to those whose infants “may be behind” (40%, 10/25). Because of the number of zero cells, it was not possible to perform statistical tests on these data.

**Table 5:** User assessment of the developmental screening tool.

User perceptions of ease of use, usefulness, and reported satisfaction			Overall (N=65), n (%)	Infants “doing well” (N=40), n (%)	Infants who “may be behind” (N=25), n (%)
**Perceived ease of use**					
	**How hard or easy was it to answer the six text messages that asked you questions about your baby?**				
		Very easy to answer the questions	38 (58)	28 (70)	10 (40)
		Easy to answer the questions	26 (40)	12 (30)	14 (56)
		Hard or very hard to answer the questions	0 (0)	0 (0)	0 (0)
		Missing response	1 (2)	0 (0)	1 (4)
	**How did you feel about how much time (the screening questions) took?**				
		The right amount of time	62 (95)	38 (95)	24 (96)
		Too much time	2 (3)	2 (5)	0 (0)
		Needed more time	0 (0)	0 (0)	0 (0)
		Missing response	1 (2)	0 (0)	1 (4)
**Perceived usefulness**					
	**Would you want to answer text questions like this again and get results about how your baby is doing when your baby is older?**				
		Yes	65 (100)	40 (100)	25 (100)
		No	0 (0)	0 (0)	0 (0)
	**Would you recommend the text messages that asked you questions about your baby to a friend with a baby?**				
		Yes	64 (97.5)	39 (97.5)	25 (100)
		Maybe	1 (2.5)	1 (2.5)	0 (0)
		No	0 (0)	0 (0)	0 (0)
**Satisfaction**					
	**Overall, how did you feel about giving answer to the 6 text messages that asked questions about your baby and getting text messages with feedback about how your baby is learning and growing?**				
		Very good, it was helpful	52 (80)	33 (83)	19 (76)
		Good, it was okay	12 (18)	6 (15)	6 (24)
		Average, it was neither good or bad	1 (2)	0 (0)	0 (0)
		Poor	0 (0)	0 (0)	0 (0)
		Unhelpful	0 (0)	0 (0)	0 (0)
	**How much did you like or dislike getting text messages with results about how your baby is doing?**				
		Liked a lot, liked getting the feedback by text	37 (57)	25 (62.5)	12 (48)
		Good, it was OK to get the feedback by text	25 (38)	15 (37.5)	10 (40)
		Neither good or bad, I didn’t care how I got the feedback	1 (2)	0 (0)	1 (4)
		Disliked, I did not like getting the feedback by text *or* totally negative, I would not want to receive feedback by text again	0 (0)	0 (0)	0 (0)
		Missing response	2 (3)	0 (0)	2 (8)

Among all participants, the time required to complete the developmental screening (ie, the time between first texting “DM” and submitting the final response) was 4 minutes and 39 seconds on an average. All but three of the phone survey participants who provided an answer (95%, 62/65) felt that the amount of time it took to answer the six text messages was “the right amount of time.” As one mother explained, “The questions were easy and I could answer them quickly.”

All but one of the mothers surveyed said they would recommend the text-based screening to a friend (97.5%, 64/65), and all mothers reported that they wanted to answer screening questions again when their baby was older (100%, 65/65).

Mothers were asked to assess the overall experience of answering the questions and getting results by text: 80% of all mothers interviewed reported that it was “very good, it was helpful” (52/65) and 18% reported that it was “good, it was okay” (12/65). Only one mother reported that it was “average,” and no mother rated it as poor or unhelpful. When specifically asked to assess the value of obtaining results of the screening by text, almost all mothers responded that they liked receiving feedback by text “a lot” (57%, 37/65) or that it was “good, OK” (38%, 25/65). Although a lower percentage of respondents whose infants “may be behind” liked receiving feedback by text “a lot” or found it “good, OK” than the respondents whose infants were “doing well,” it was not possible to calculate statistics due to the number of cells with zero data.

## Discussion

### Principal Findings

The primary aims of this pilot study were to assess the usability and acceptability of a validated interactive text message-based tool for developmental screening of infants of low-income mothers as a strategy to make developmental screening more broadly available, particularly among low-income populations.

This pilot study found that it was feasible to create an interactive text-based screening tool based on a validated instrument, and low-income mothers of infants aged 8-10 months found the tool both usable and acceptable. The findings of this study are important because the ubiquity of mobile phones (100%) [10] and texting (95%) [11] among Americans aged 18-29 years creates the potential to make screening widely available to parents of infants, including those from low-income households and on Medicaid who are at a heightened risk of developmental delays especially in the early years.

### Usability and Acceptability

Despite a long history of theory-based approaches for assessing the usability and acceptability of web interfaces, there is a paucity of literature on mHealth usability and the inherent challenges of mHealth usability assessments such as small screens and the lack of software that captures physical interactions with devices [19]. There are perhaps even greater challenges in capturing user experience in a text intervention. For example, users own a variety of devices from basic mobile phones to feature-rich mobile phones of varying sizes, and the screening tool may appear quite different depending on the device used. In addition, devices and platforms capture very limited data on interactivity beyond the time and content of interactive messages sent and received.

This study therefore focused on practical measures of performance reflecting actual use of the intervention and user assessments of their experience. The study found no differences in any of the measures of usability according to the infants’ developmental status or any of the demographic or socioeconomic characteristics. All mothers were able to respond to all questions and complete the screening (100%, 81/81) including the 21% of mothers who wanted to change one or more responses (17/81). Challenges due to poor mobile coverage in one of the clinics did not prevent participants from successfully completing the screening.

With regard to acceptability, mothers, including those who received a report that their infant “may be behind,” valued the tool as well as the experience of using it and receiving results by text. Almost all mothers ranked the experience “very good” or “good” and all but one said that they would recommend it to a friend. All mothers in the study expressed the desire to repeat the screening in the future when their infants were older.

There were several lessons learned that should be considered in future iterations and research. First, although the pilot study focused on infants aged 8-10 months, future research and implementation should extend the tool to cover additional ages recommended by AAP and Medicaid’s Child Core Set of health quality measures [4-6]. Second, words matter. The wording of one of the screening questions was associated with the highest error rates and should be reviewed and tested. Third, although there was no difference in the measures of usability for Spanish and English speakers in this pilot study, future iterations should offer a choice of language to the users. Fourth, the observation that mothers whose infants “may be behind” were even less likely to report a previous screening suggests that this approach could potentially reach and identify infants at the highest risk of developmental delay, who do not have a medical home and who might otherwise not be identified in a timely way. Fifth, a critical concern in conducting a developmental screening is how to refer mothers whose infants “may be behind” for further screening and, if indicated, diagnosis and treatment for their infants as required and supported by the Early and Periodic Screening, Diagnostic and Treatment benefit of Medicaid [6]. The ability of the ITP representative to reach 93% (28/30) of mothers whose infants “may be behind” in person in the clinic or by phone for possible follow-up was therefore an important aspect of the study process. Clearly, this is only the first, albeit necessary, step to ensure that at-risk infants receive the required diagnosis and appropriate treatment in a timely manner.

### Implementation and Evaluation Approaches

To our knowledge, this is the first study to use texting of a validated tool to conduct developmental screening in the United States and to assess such a tool with a low-income population largely enrolled in Medicaid. It is therefore important to incorporate the lessons learned in this study about usability and acceptability and test the effectiveness of this approach on a larger scale including in a randomized clinical trial [24]. Although many mHealth studies do not include an implementation component [15], this study was designed with a view toward large-scale implementation and evaluation.

The combination of broadly available mobile technology, a valid screen, and the partnerships put in place for this study—with *Text4baby*, the State of Maryland WIC program, and Prince George’s County ITP program—provides a potential model for scaling up and further evaluation of an approach to promote widespread access to developmental screening and potentially reach at-risk populations. Largescale mobile health programs such as *Text4baby* could make the developmental screening tool available to mothers enrolled in that program where follow-up is available from an infant’s pediatrician or an early intervention program such as ITP. To connect parents of infants who “may be behind” to follow-up, this study suggests that phone calls, in this study’s case, from the ITP to parents, following screening may be an effective method.

The text-based screening tool could also be used and evaluated in a medical home or other care setting. As noted in a recent review of mobile health applications to promote early language development, “m-Health could provide a natural extension of interventions and messages delivered within the primary care setting” [9]. For example, the waiting room staff could ask a parent to trigger the screening tool by sending a text message to a designated number. The parent could then receive and review the results immediately with the infant’s pediatrician or another provider.

In both cases, there could be advantages of embedding developmental screening in a broader texting program such as *Text4baby*. Parents could be sent additional text messages with information about resources for child development and tailored parenting tips as well as reminders to attend well-baby visits, a strategy that has been shown to be effective in increasing attendance rates [25].

### Limitations and Strengths

Limitations of this pilot study include its relatively small sample size and primary focus on descriptive data. Participants recruited from two WIC clinics located near a major metropolitan area had higher educational levels than the United States average levels [26]. Maryland also has one of the highest rates of developmental screening in the United States [1]. Study participants may not reflect the overall population of low-income mothers of infants in this age cohort, given that more than half the mothers reported that their infants had had an earlier developmental screening. As part of explaining the purpose of the study and assuring that participant consent was “informed,” the study team likely raised awareness of developmental domains beyond the information provided in the screening messages. In addition, this pilot study did not include an independent test of validity of the text version as compared to a more traditional format.

The study has several important strengths. Because of the partnership with WIC and its clinics in Prince George’s County, the study was able to reach and recruit low-income mothers whose infants may be at elevated risk for developmental delays. Due to the partnership with ITP, it was possible to reach and offer follow-up to mothers whose infants “may be behind.” The study also focused on user experience and input in the early stages of development and testing of an innovative mHealth tool with lessons that should be incorporated in future implementation and research.

### Conclusions

This pilot study addresses the low rates of developmental screening among low-income populations in the United States by investigating how to build on the dramatic growth in the coverage of mobile technology and the broad use of texting within this population.

The study concluded that a validated developmental screening tool delivered by text message to mobile phones is both usable and acceptable by low-income mothers of infants. The results and lessons learned in this study can inform further evaluation with larger sample sizes and at other recommended ages. It also offers insights for potential models for scalable implementation of validated developmental screening using text messaging to screen and support mothers of infants.

The combination of broadly available mobile technology and the partnerships put in place for this study with *Text4baby,* the State of Maryland’s WIC program, and Prince George’s County ITP program holds promise for scaling up and further evaluating a model to promote more widespread developmental screening.

This study contributes to the growing body of evidence supporting the feasibility as well as the effectiveness of using text messaging to address important health outcomes and disparities. Studies have documented the impact of text messaging programs on health knowledge, appointment attendance, vaccination compliance, and other health behaviors. The findings of this study extend mHealth research to support developmental screening and address services needed by at-risk populations.

## Appendix

Multimedia Appendix 1 Summary of developmental screening tools [6].

## Abbreviations

AAP: American Academy of Pediatrics
ITP: Infants and Toddlers Program
MDHMH: Maryland Department of Health and Mental Hygiene
mHealth: mobile health
PEDS:DM: Parents’ Evaluation of Developmental Status: Developmental Milestones
WIC: Women, Infants and Children Program
