# Supplementary material for: Usability and Acceptability of a Text Message-Based Developmental Screening Tool for Young Children: Pilot Study
Source: JMIR Pediatr Parent. 2019 Jan 30;2(1):e10814. doi: 10.2196/10814 (PMC6716482; doi:10.2196/10814)
Supplement: Multimedia Appendix 1 [file pediatrics_v2i1e10814_app1.pdf]

Multimedia Appendix 1: Summary of Review of Developmental Screening Tools [6]

Tool	Parental Report	# of Questions	All Domains	Sensitivity & Specificity	Age Range	Scoring	Time Burden	Reading Level
Ages & Stages	✓	30	✓	➢ 70%	4-60 months	Pass/ fail	15-30 minutes	3rd-12th grade
PEDS	✓	10	✓	➢ 70%	0-95 months	Risk level	2-10 minutes	4th-5th grade
PEDS: DM	✓	6-8	✓	➢ 70%	0-95 months	Pass/ fail	3-5 minutes	< 2nd grade
